# Electron spin coherence near room temperature in magnetic quantum dots

**DOI:** 10.1038/srep10855

**Published:** 2015-06-04

**Authors:** Fabrizio Moro, Lyudmila Turyanska, James Wilman, Alistair J. Fielding, Michael W. Fay, Josef Granwehr, Amalia Patanè

**Affiliations:** 1 School of Physics and Astronomy, The University of Nottingham, University Park, Nottingham NG7 2RD, UK; 2 School of Chemistry and Photon Science Institute, The University of Manchester, Oxford Road, Manchester M13 9PL, UK; 3 Nottingham Nanotechnology and Nanoscience Centre, University Park, Nottingham NG7 2RD, UK; 4 Institute of Energy and Climate Research (IEK-9), Forschungszentrum Juelich, Juelich D-52425, Germany

## Abstract

We report on an example of confined magnetic ions with long spin coherence near room temperature. This was achieved by confining single Mn^2+^ spins in colloidal semiconductor quantum dots (QDs) and by dispersing the QDs in a proton-spin free matrix. The controlled suppression of Mn–Mn interactions and minimization of Mn–nuclear spin dipolar interactions result in unprecedentedly long phase memory (*T*_M_ ~ 8 μs) and spin–lattice relaxation (*T*_1_ ~ 10 ms) time constants for Mn^2+^ ions at *T* = 4.5 K, and in electron spin coherence observable near room temperature (*T*_M_ ~ 1 μs).

The controlled incorporation of magnetic impurities in a semiconductor provides a means to manipulate magnetic and electronic interactions, one of the exciting challenges in modern condensed matter systems^1^. In particular, the interactions between magnetic ions and a host material constrained to low dimensionality provide an additional degree of freedom in tailoring physical properties and a route to the discovery of phenomena of fundamental and technological interest^2^,^3^,^4^,^5^,^6^.

A promising way of investigating confined electron spins is through colloidal quantum dots (QDs)^7^. These nanocrystals consist of semiconductor materials (e.g. ZnO, PbS, CdSe) surrounded by organic capping ligands or inorganic shells acting as a dielectric insulating barrier between individual nanostructures and facilitating solubilization of QDs in different solvents. Recent advances in chemical synthesis have enabled the controlled incorporation of magnetic impurities with concentration down to a single impurity per QD^8^, and added flexibility in the manipulation of the QD surface and environment^9^,^10^ to create multi-functional devices^11^,^12^,^13^.

Among transition metal ions, Mn^2+^ has received long-standing interest as a dopant in semiconductors^14^ because of its large spin magnetic moment (*S* = 5/2) and quenched orbital magnetic moment (*L* = 0). The latter implies reduced electron spin*–*lattice interactions, leading to a relatively long electron spin–lattice relaxation time constant (*T*_1_)^15^, as well as to an efficient energy transfer between Mn spins and confined quantum carriers mediated by *sp–d* interactions^16^,^17^. Recently, the interest in Mn-doped QDs has risen due to the observation of Rabi oscillations and quantum coherence with a phase memory time constant *T*_M_ of the order of a few microseconds at liquid He-temperature^7^,^18^,^19^,^20^. These exceed the coherence times previously reported for layered^21^, quantum wells^22^,^23^ and self-assembled QDs^20^,^24^,^25^, either doped with Mn ions or confining a single electron, by one order of magnitude or more. This result can be ascribed to the localization of electrons in 3*d* orbitals, small spin–orbit interactions and minimized nuclear spin bath noise. It has also been suggested that the dielectric solvent and organic ligands in colloidal QDs effectively screen Mn–Mn dipolar interactions^7^. Similar characteristics are present in molecule-based systems where quantum coherence in the range of microseconds has been reported^26^,^27^,^28^,^29^. Longer electron spin quantum coherence times were reported for phospourous donors in isotopically purified silicon^30^ (i.e. *T*_M_ ~ 1 ms), although at *T* = 100 mK. A remarkable example is represented by nitrogen vacancy (NV) centres in diamond where quantum coherence is observed in the millisecond range at room temperature^31^ because of the atomic-like localization of NV centres, low mass of carbon atoms, which suppress spin-orbit interactions, and isotopically purified host nuclei.

Pulsed electron spin resonance (ESR) studies have enabled the identification of the main sources of electron spin dephasing in magnetic colloidal QDs, i.e. Mn*–*Mn dipolar interactions and hyperfine interactions of the Mn spins with the protons of the capping ligands^7^,^19^. These findings indicate that much longer electron spin dynamics and improved control of quantum coherences could be achieved by tailoring the separation between the Mn ions and by reducing Mn–nuclear spin interactions. To the best of our knowledge such potential has not yet been explored in QDs and may enable significant advances in nanoscience and quantum technologies.

In this work we isolate and spatially confine Mn^2+^ ions by dispersing colloidal PbS:Mn QDs in a diamagnetic, proton-spin free matrix, thus resulting in a controlled suppression of Mn–Mn dipolar interactions in the QD ensemble and reduced interactions with the nuclear spins surrounding the QDs. The isovalence of Mn^2+^ and Pb^2+^ atoms ensures that the Mn-doped PbS QDs are electrically neutral and that Mn–Mn interactions mediated by free electrons (i.e. RKKY) are absent^32^. Our pulsed ESR experiments show that such Mn spins possess unprecedentedly long phase memory time and spin–lattice relaxation time constants. Most importantly, this long electron spin dynamics could be observed near ambient temperature, opening up realistic scenarios for further investigations and exploitation of carrier*–*Mn^2+^ magnetic interactions in quantum confined systems.

## Results

### Materials

**Figure 1:**
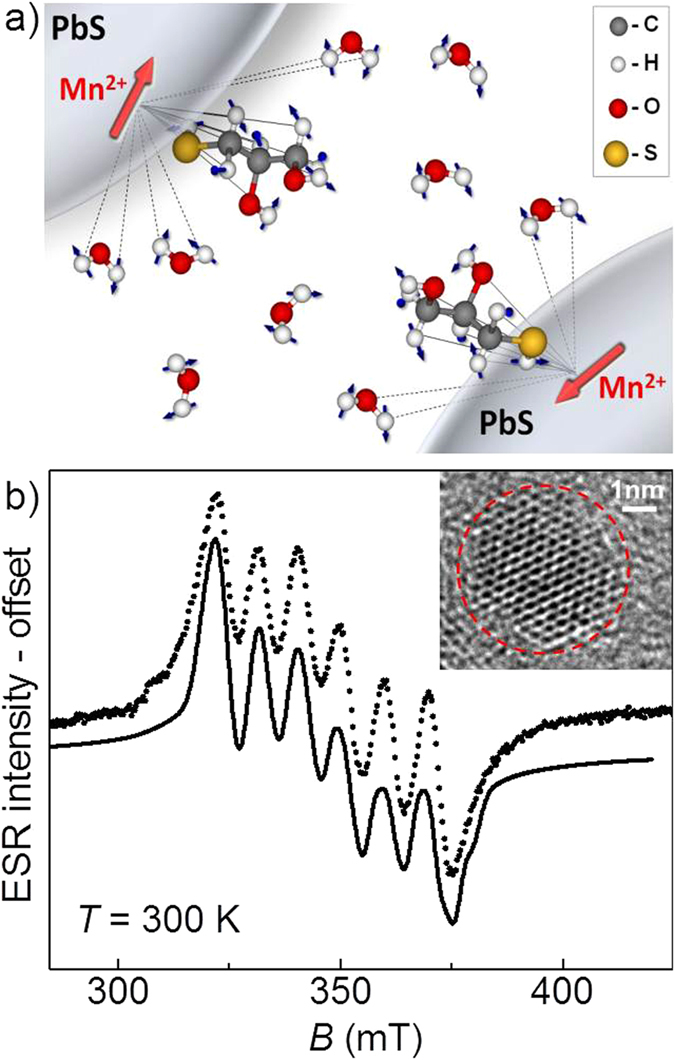
Magnetic interactions in diluted QDs and physical characterization. (**a**) Representation of magnetic interactions between Mn ions (red arrows) in two neighbouring PbS QDs dispersed in water. The hyperfine interactions between Mn spins and proton nuclear spins of the capping ligands and solvent matrix are shown. The continuous lines indicate strong interactions while dotted lines indicate weak interactions. The ^207^Pb nuclear spins are not shown. (**b**) X-band CW-ESR spectrum (dotted line) for powder sample of **Mn**_**0.05%**_ and simulation (continuous line) to a spin-Hamiltonian model (see text). Inset: High resolution TEM image of **Mn**_**0.05%**_. The dashed red line sketches the boundary of the QD.

Colloidal PbS:Mn QDs capped with thioglycerol/dithiolglycerol ligands, Fig. 1(a), were synthesised in aqueous solution^33^ with Mn weight content *x* = 0.05% (sample **Mn**_**0.05%**_) and *x* = 0.01% (sample **Mn**_**0.01%**_), corresponding to a nominal average Mn ion per QD ratio of 1:2 and 1:10, respectively. The synthesis of PbS:Mn QDs in 99.8% deuterated water (sample **DMn**_**0.05%**_) produces a sample free from proton-spin solvent molecules. All QDs were studied as powders and as frozen solutions in H_2_O and in D_2_O, as well as in 1:1 mixtures of H_2_O:C_3_H_8_O_3_ (glycerol-H_8_) and D_2_O:C_3_D_8_O_3_ (glycerol-D_8_).

### Continuous–wave ESR

Figure 1(b) shows the CW-ESR spectrum at X-band frequency (ν_mw_ = 9.8 GHz) for powder **Mn**_**0.05%**_ at *T* = 300 K. The spectrum consists of six lines centred close to the free electron *g* value. We ascribe the six features to the six hyperfine lines of ^55^Mn nuclei (*I* = 5/2) interacting with the *d*-electrons of Mn^2+^ (*S* = 5/2)^34^. From the fit of the CW-ESR spectrum, shown in Fig. 1(b), it was possible to determine the isotropic spin Hamiltonian parameters^34^ *g* = 2.001 ± 0.005 and *A* = 267 ± 1 MHz which were consistent with that reported previously^19^,^35^. For the fit an intrinsic Lorentzian linewidth *Γ_*L*_* = 0.5 ± 0.1 mT was assumed. Further constraints to these parameters were obtained by fitting a CW-ESR spectrum at W-band frequency (ν_mw_ ~ 94 GHz, see Supplementary Fig. S1). The zero-field splitting parameter, *D*, could not be quantified unequivocally because of the large linewidth broadening most likely caused by large strain of the spin-Hamiltonian parameters. In Fig. 1(b) we report an attempt with *D* = 50 MHz and an overall *g-*, *A-* and *D-* strain, 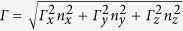 = 11.78 mT, where 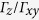 = 3:1 and 
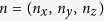
 defines the orientation of the magnetic field vector. The small magnetic anisotropy, *D*, and large anisotropic strain suggest that Mn ions are surrounded by a distribution of distorted cubic environments, possibly due to their proximity to the QD surface.

### Mn–Mn dipolar interactions

**Figure 2:**
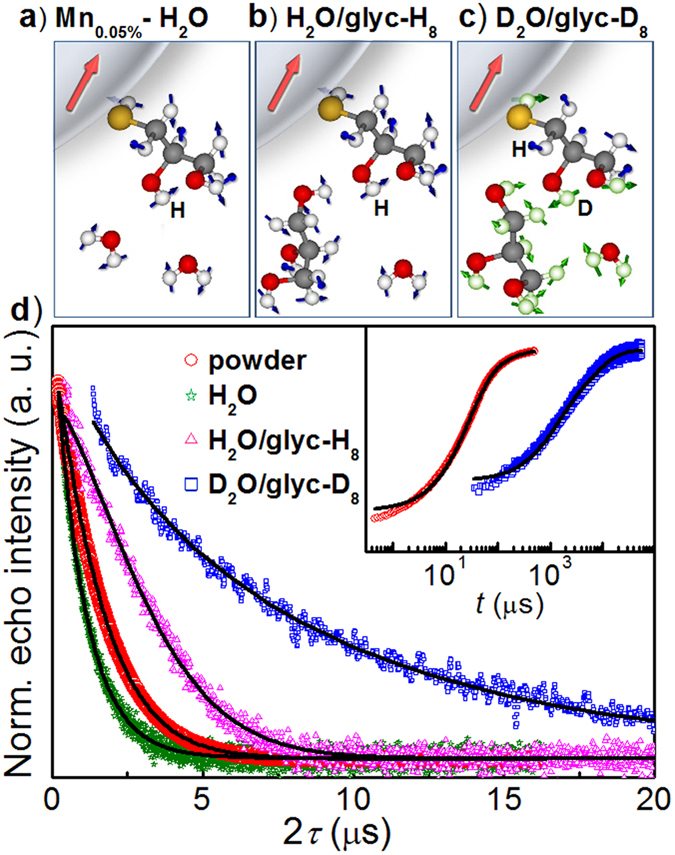
Spin dynamics for quantum dots in different matrices. Representation of QDs in different matrix solvents: (**a**) H_2_O, (**b**) H_2_O/glycerol-H_8_ and (**c**) D_2_O/glycerol-D_8_. Green circles and arrows represent deuterium atoms and spins, respectively. (d) Hahn echo decay for **Mn**_**0.05%**_ as powder and frozen solutions at *T* = 5 K. Black lines are fits to equation (2). Inset: Inversion recovery echo traces for powder (red circles) and frozen solution in D_2_O/glycerol-D_8_ (blue squares) of **Mn**_**0.05%**_ QDs along with the fits to equation (3) (black line).

We now examine the effect of the Mn-Mn separation in PbS:Mn QDs diluted in different solvents (Fig. 2a–c) on the phase memory and spin–lattice relaxation time constants of Mn^2+^ ions at *T* = 5 K (see Fig. 2(d) and Table 1). For **Mn**_**0.05%**_ QDs as powder (see red curve in Fig. 2(d)), the fit of the spin echo decay to a stretched exponential decay function gives *T*_M_ ~ 1.6 μs, while the fit of the inversion recovery echo signal to a bi-exponential function gives *T*_1_ ~ 130 μs and *T*_SD_ ~ 27 μs. The fast relaxing contribution, *T*_SD_, is ascribed to spectral diffusion and therefore will not be discussed in the following^36^. In the **Mn**_**0.05%**_ powder sample the average Mn-Mn distance is *d* ~ 6 nm^19^. Using a simple model for two interacting spins, we estimate a maximum dipolar field, *B*_dip_, experienced by next neighbour Mn ions at such a distance of *B*_dip_ ~ 50 μT, corresponding to a dipolar time constant *T*_dip_ ~ 1 μs. These values suggest that magnetic dipolar interactions between Mn ions are an important source of electron spin dephasing.

To increase the Mn-Mn separation, we disperse the QDs in aqueous solution (Fig. 2a) with density *δ* = 5 mg/ml, corresponding to an average distance between the Mn^2+^ ions *d* ~ 35 nm. Thus, we estimate *B*_dip_ ~ 0.2 μT with an upper bound for *T*_M_ given by *T*_dip_ ~ 60 μs. Surprisingly, the resulting spin echo decay (see green curve in Fig. 2(d)) shows a faster relaxation, *T*_M_ ~ 1.0 μs at *T* = 5 K, compared to that of the powder. The same effect was observed in deuterated water (Supplementary Fig. S2). Such fast spin echo decay is likely due to a combination of several factors: the formation of regions with high QD concentrations resulting from the crystallization of water^29^, the presence of solvent protons at a short distance from the Mn^2+^, and the absorption of microwave radiation by the water molecules, which leads to enhanced vibrations and librations^37^ of the dielectric dipoles and heating of the environment. To overcome these effects, we dilute the QDs in H_2_O/glycerol-H_8_ (Fig. 2(b)). Addition of glycerol to aqueous solutions produces a glassy matrix, which reduces lattice vibrations and QD agglomeration^38^. As shown in Fig. 2(d) (see magenta curve), in this case we achieved a significantly longer spin-echo decay (*T*_M_ ~ 3.5 μs) compared to both **Mn**_**0.05%**_ QDs as powder and dispersed in water. The further reduction of the Mn concentration in the QDs to *x* = 0.01% in frozen H_2_O/glycerol-H_8_ mixture did not lead to significant changes in the spin echo decay (Supplementary Fig. S3 and Table SI), thus proving that we have reached a limit where the spatial separation between the QDs is large enough to suppress Mn*–*Mn dipolar interactions.

### Nuclear spin bath dephasing

The suppression of Mn*–*Mn dipolar interactions enables us to identify other sources of electron spin dephasing. In particular, protons present in the water solvent can dephase electron spins via nuclear spin flip-flop (i.e spin diffusion) and nuclear motions (i.e. rotational diffusion and vibration processes)^29^ (see Fig. 1(a)). The dilution of QDs in deuterated water and glycerol (Fig. 2(c)) should lead to a longer *T*_M_ because the electron–nuclear spin coupling is diminished by the smaller magnetic moment of D-nuclei compared to H, μ(D)/μ(H) = 0.307, and by the smaller nuclear spin diffusion effects, which scale as the square of the nuclear magnetic moment. Overall *T*_M_ is expected to increase approximately with the negative third power of the nuclear moment^29^, *μ*^−3^, corresponding to a factor of 35. Our spin echo decay and inversion recovery data (blue curves in Fig. 2(c)) show that the spin dynamics of **DMn**_**0.05%**_ dispersed in D_2_O/glycerol-D_8_ is longer (*T*_M_ ~ 8 μs and *T*_1_ ~ 8 ms at *T* = 5 K) compared to that for **Mn**_**0.05%**_ in H_2_O/glycerol-H_8_. Furthermore, we find that *T*_1_ is increased by a factor of ~80 compared to **Mn**_**0.05%**_in powder, thus suggesting that spin–lattice relaxation processes are mediated by Mn–Mn and Mn–nuclear spin bath interactions.

### Electron–nuclear interactions

**Figure 3:**
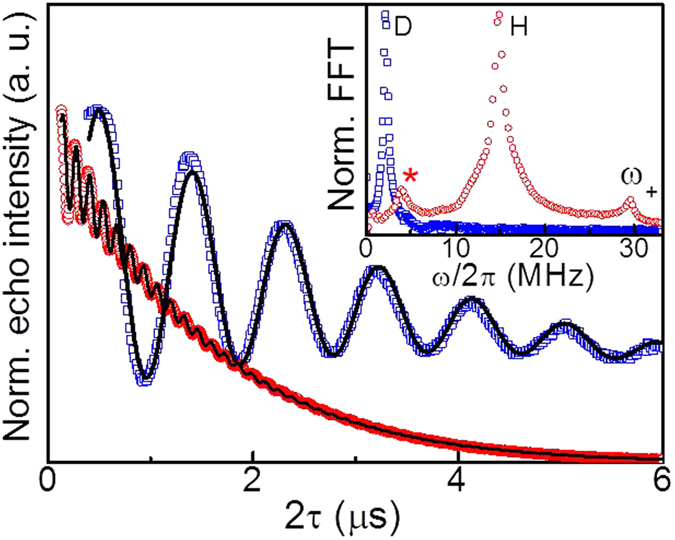
Electron–nuclear spin dynamics. 2p-ESEEM traces of **Mn**_**0.05%**_ powder (red circles) and **DMn**_**0.05%**_ frozen solution in D_2_O/glycerol-D_8_ (blue squares) at *T* = 5 K and their Fast Fourier Transform (inset). Black lines are simulations by equation (4). For **Mn**_**0.05%**_ we observe a small peak at *ω*_*I*_/2*π* ~ 3.9 MHz indicated with the symbol * which is very close to the Larmor frequency of ^23^Na (*ω*_*I*_/2*π* = 3.88 MHz, *μ* = 2.22 *μ*_N_, and natural abundance ~100%). The presence of ^23^Na nuclei could be due to the use of Na salts in the synthesis and possibly incorporated as interstitial impurity within the PbS nanocrystals^45^. Alternatively, coupling to ^207^Pb could be the cause of this peak. We also observe a weak peak at *ω*/2*π* = 29.5 MHz, which we ascribe to sum, *ω*_+_ = *ω*_*α*_ + *ω*_*β*_, harmonic of the principal proton frequencies, *ω*_*I*_ ≈ *ω*_*α*_ ≈ *ω*_*β*_, resulting from Mn*–*proton spin dipolar interactions^36^.

To identify the nuclear species responsible for the electron spin dephasing, we have performed 2-pulse electron spin echo envelope modulation (2p-ESEEM) experiments on powder **Mn**_**0.05%**_and on **DMn**_**0.05%**_ in D_2_O/glycerol-D_8_ (Fig. 3). The 2p-ESEEM data were fitted to a modulated stretched exponential function. For **Mn**_**0.05%**_ the modulated part of the echo decay is dominated by a contribution with a shorter period than for **DMn**_**0.05%**_. The Fast Fourier Transform (FFT) of the data shows intense peaks at *ω*_*I*_/2*π* ~ 14.9 MHz for **Mn**_**0.05%**_ and *ω*_*I*_/2*π* ~ 2.3 MHz for **DMn**_**0.05%**_ QDs (see inset in Fig. 3), which are close to the natural Larmor frequencies of hydrogen (*ω*_*I*_/2*π* = 14.69 MHz) and deuterium (*ω*_*I*_/2*π* = 2.25 MHz) at *B* = 345 mT, respectively. The observation of Mn–deuterium ESEEM for **DMn**_**0.05%**_can be attributed to the proximity of deuterated solvent molecules to Mn ions near the QD surface. On the other hand, the apparent absence of the modulations at the hydrogen Larmor frequency may be ascribed to the partial exchange between D_2_O and hydrogens of the O–H and S–H groups in the capping ligands (see Fig. 2(c)). We note that despite the relatively large natural abundance of ^207^Pb nuclei (~22%) their contribution to the ESEEM spectra (*ω*_*I*_/2*π* = 3.08 MHz) could not be unambiguously assigned^19^.

The ESEEM modulation depth, *k*, depends on the electron–nuclei distance as well as on the nuclear spin density in the proximity of the electron spins^36^. We find that *k* is essentially unchanged for powder and corresponding frozen solution (see Table 1), suggesting a similar nuclear spin density for both samples (see Fig. 1(a)).

### Spin dynamics temperature dependence

**Figure 4:**
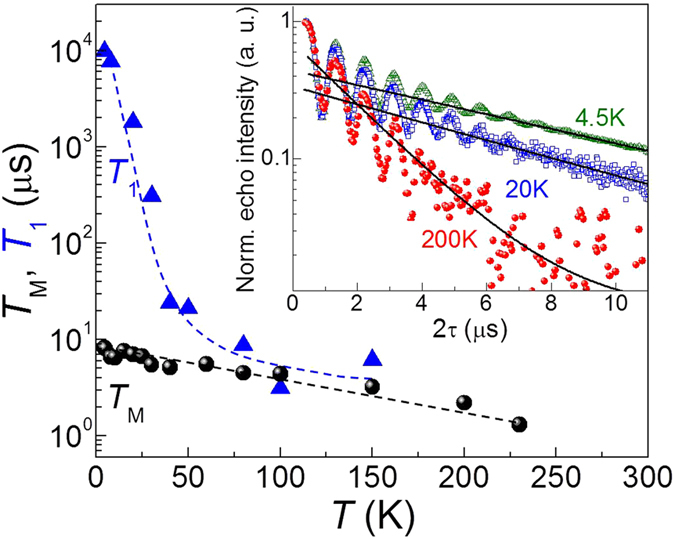
Temperature dependent spin dynamics. Temperature dependence for *T*_M_ (spheres) and *T*_1_ (triangles) for **Mn**_**0.05%**_ in D_2_O/glycerol-D_8_. Dashed lines are guides for the eye. Inset: representative spin echo decay traces at *T* = 4.5 K (green), 20 K (blue) and 200 K (red) along with the fitting to a mono-exponential decay function (black line).

Long lifetimes of Mn^2^^+^ spins in PbS QDs are observed at *T* > 5 K. Figure 4 shows the temperature dependence of *T*_M_ and *T*_1_ for **Mn**_**0.05%**_ QDs in D_2_O/glycerol-D_8_ (Supplementary Table SII). For *T *< 20 K, *T*_M_ is essentially constant while at higher temperature *T*_M_ smoothly decreases, reaching *T*_M_ ~ 1.0 μs at *T* = 230 K (Fig. 4). For *T* > 230 K the echo intensity is comparable to the noise level, preventing an estimation of *T*_M_. The stretching parameter *s* remains constant at *s* = 1 across the entire temperature range investigated. For *T* < 80 K, *T*_1_ is much larger than *T*_M_ and depends strongly on temperature, with *T*_1_ ~ 10 ms at 4.5 K and *T*_1_ ~ 9 μs at 80 K. For *T* > 80 K, *T*_1_ ~ *T*_M_ and its temperature dependence is weaker.

## Discussion

Our study indicates that the relaxation properties of Mn spins encapsulated into PbS colloidal QDs can be tailored by modifying the environment of the QDs. By dispersing the QDs in a glassy matrix that is free of protons, we have suppressed the major sources of electron spin dephasing, i.e. Mn–Mn dipolar interactions, and minimized the interactions of the Mn ions with the nuclear spin bath. As a result, we have achieved an enhancement of the phase memory and the spin–lattice relaxation time constants by a factor of ten, and we have observed spin coherence near room temperature. This was possible due to the large separation between the Mn ions (*d* ~ 35 nm) and the small magnetic moment of deuterated matrix molecules, which reduce the time dependent magnetic field perturbations seen by each individual Mn ion due to the surrounding electron and nuclear spins.

In addition, our results show that in the temperature regime below 20 K, where *T*_1_ >> *T*_M_ and 1/*T*_M_ ~ constant, spin–lattice relaxation processes are not a limiting factor for the electron spin coherence. Instead, our 2p-ESEEM experiments indicate that electron–deuterium spin interactions represent a source of electron spin dephasing. The fact that *s* = 1 across the entire temperature range investigated, suggests that nuclear spin diffusion processes are a less important source for electron spin dephasing in deuterated solution than for **Mn**_**0.05%**_ in H_2_O/glycerol-H_8_. In the latter *T*_M_ and *s* are temperature dependent with *s* > 1 for *T* < 20 K (Supplementary Fig. S4). We ascribe this effect to the smaller magnetic moment of deuterium compared to that of protons^25^,^26^.

In the temperature regime above 80 K, where *T*_1_ ~ *T*_M_, spin–lattice relaxation processes begin to dominate the electron spin echo dephasing via enhanced thermal motion of the nuclear spins of the capping ligands and/or of the solvent molecules near the QD surface. This is likely due to the softening of the glassy matrix approaching the melting point. These motions modulate Mn–nuclear spin dipolar interactions, leading to electron spin dephasing and rapid exchange of magnetic energy between the Mn^2+^ spins and its environment.

In summary, we have demonstrated quantum coherence near room temperature for electrons spins confined in colloidal quantum dots. The long electron spin dynamics lifetime observed at *T* = 4.5 K (*T*_M_ ~ 8 μs and *T*_1_ ~ 10 ms) and, most importantly, the observation of quantum coherence up to *T* = 230 K (*T*_M_ ~ 1 μs) are unprecedented for Mn ions and very rare amongst transition metal ions. For comparison, phase memory or spin–spin relaxation (*T*_2_) times of Mn spins or confined electrons in other low dimensional systems, such as self-assembled QDs^20^,^24^,^25^, layered magnetic semiconductors^21^ and quantum wells^22^,^23^, do not exceed 1 ns, and those for magnetic colloidal QDs in the solid state are <1 μs^7^,^39^. In addition, we note that *T*_1_ for **Mn**_**0.05%**_ is one order of magnitude longer than that found in self-assembled QDs^15^ and diluted magnetic quantum wells^17^,^40^. Overall, the *T*_M_ and *T*_1_ values found for PbS:Mn QDs are comparable only to molecules based on Cr and V ions in D_2_O/glycerol-D_8_^41^ and on Cu ions diluted in a diamagnetic matrix^28^. Considering that further improvements of the Mn spin lifetime could be achieved by incorporation into nuclear spin free nanocrystals, by deuteration of the capping ligands, by substitution of the ligands with larger steric hindrance^42^, and by embedding the QDs in a nuclear-spin free matrix rigid at room temperature, colloidal QDs could enable the exploitation of magnetic interactions in confined electron spins for spintronics and quantum information processing applications.

## Methods

### Transmission electron microscopy

Transmission electron microscopy (TEM) images of PbS:Mn QDs deposited on a graphene oxide-coated grid were recorded on a JEOL 2100F microscope operating at 120 kV. The TEM study shows that the QDs have the rock-salt crystal structure of bulk PbS and an average core diameter *φ* = 4.5 ± 1.2 nm (inset in Fig. 1(b)).

### Samples preparation

Powder samples for ESR experiments were freeze dried overnight and inserted into 3 mm outer diameter quartz tubes. Then, the tubes were flushed with nitrogen gas to remove moisture and oxygen contamination and closed with stop cocks. Solution samples were injected into 4 mm outer diameter quartz tubes from sealed vials. The tubes were then closed with stop cocks and frozen in liquid nitrogen before insertion in the ESR resonator which was precooled at 5 K.

### Electron spin resonance

Pulsed and continuous-wave (CW) ESR experiments were performed on a Bruker ElexSys E580 spectrometer coupled to a dielectric resonator (MD5), and additional CW-ESR experiments were performed on a Bruker EMXmicro spectrometer coupled to a Super High-Q cavity. Both spectrometers operate at X-band frequency (ν_mw_ = 9.8 GHz). CW-ESR spectra were recorded with magnetic field modulation amplitude and frequency of 0.1 mT and 100 kHz, respectively. The W-band CW-ESR spectra were recorded on a home-built spectrometer based on a Krymov bridge and probe^43^, operating at a frequency ν_mw_ = 94.90 GHz, with a modulation amplitude of 0.1 mT and modulation frequency of 10 kHz.

### ESR simulation and data analysis

The simulation of the CW-ESR spectra in Fig. S1 were performed with the Easyspin toolbox^44^ using the spin-Hamiltonian model^34^:
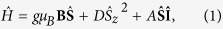
where *g* is the Landé *g* factor, *μ*_*B*_ is the Bohr magneton, **B** is the magnetic field vector, *D* and *E* are the axial and planar magnetic anisotropy, *A* is the isotropic hyperfine coupling constant, and *S* and *I* are the electron and nuclear spin quantum numbers, respectively. The first, second and third terms account for the Zeeman interaction, the zero-field splitting, and the hyperfine interaction, respectively. To simulate *D* strains, zero field interactions of rhombic symmetry were assumed.

Echo field swept ESR spectra (Supplementary Fig. S5) were recorded at *T* = 5 K with a primary echo sequence, *π*/2 − *τ − π − τ − echo* with *π* = 32 ns, *τ* = 200 ns, and a shot repetition time of 1048 μs. Spin echo decay experiments were carried out by increasing the inter-pulse delay, *τ*, of the primary echo sequence. Microwave pulse lengths of *π* = 120 ns and 600 ns were used to suppress proton and deuterium electron spin modulations, respectively. The phase memory time *T*_M_ was estimated from fitting the spin-echo signal (*I*) with the function:
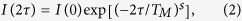
where *s* is a stretching parameter.

The inversion–recovery pulse sequence, *π *− *t *− *π/2* *−* *τ* − *π −* *τ −* *echo*, was recorded with *π* = 32 ns, *τ* = 0.2 μs and variable *t*. The spin–lattice relaxation time constant, *T*_1_, was estimated by fitting the signal with the function:
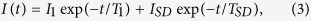
where *I*_1_ and *I*_SD_ are amplitudes, and *T*_SD_ is the spectral diffusion time constant.

Two-pulse electron spin echo envelope modulation (2p-ESEEM) experiments were performed by fixing the microwave pulse length to *π* = 32 ns, and changing the delay between the microwave pulses of the primary echo sequence. The results were simulated with the function:
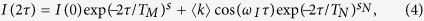
where 〈*k*〉 is the modulation depth, *ω*_*I*_ is the Larmor angular frequency of a nucleus coupled to the electron spin, *T*_N_ and *s*_N_ are the decay constant and stretching parameter of the electron–nuclear spin oscillations, respectively. All the pulsed-ESR experiments were conducted at *B* = 345 mT, which corresponds to the maximum echo intensity of the EFS spectrum (Supplementary Fig. S5).

The fits of equations (1, 2, 3, 4) to the spin echo decay and inversion recovery traces (Supplementary Fig. S6) were done by using the fitting routine implemented in Origin 8.0. The results of the fits are reported in Table 1.

### Dipolar time constant, *T*_dip_

From the dipolar frequency, *ω*_dip_/2*π* = 1/*T*_dip_^36^, for two *S*_*A*_ and *S*_*B*_ electron spins at distance *r*, we obtain:
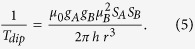


## Additional Information

**How to cite this article**: Moro, F. *et al.* Electron spin coherence near room temperature in magnetic quantum dots. *Sci. Rep.* **5**, 10855; doi: 10.1038/srep10855 (2015).

## Supplementary Material

Supplementary Information

## Figures and Tables

**Table 1:** **Fitting parameters for spin echo, inversion recovery and 2p-ESEEM traces.** Results of the fittings of the spin echo decay and inversion recovery data (see Fig. 2) by equations (2) and (3), respectively, and of the simulations for the 2p-ESEEM data (see Fig. 3) by equation (4) with 5% error bars.

**Mn**_**0.05%**_	**π (ns)**	**τ (ns)**	***T***_**M**_ **(μs)**	***s***	***T***_**N**_ **(μs)**	***s***_**N**_	***k***	***T***_**1**_ **(μs)**	***T***_**SD**_**(μs)**
**Powder**	32	130	1.56 ± 0.01	1.13 ± 0.01	0.50	1.0	0.12	130 ± 10	27 ± 5
	120	210	1.69 ± 0.01	1.13 ± 0.01	-	-	-	-	-
**H**_**2**_**O**	120	210	0.99 ± 0.01	0.96 ± 0.01	-	-	-	-	-
**H**_**2**_**O/glyc.-H**_**8**_	32	400	3.54 ± 0.02	1.51 ± 0.02	1.5	0.9	0.09	-	-
**D**_**2**_**O/glyc.-D**_**8**_	32	400	8.4 ± 0.2	1.0 ± 0.1	2.4	1.3	0.52	7670 ± 70	1230 ± 10
	600	1362	8.45 ± 0.06	-	-	-	-	-	-
