# Supplementary material for: Electron spin coherence near room temperature in magnetic quantum dots
Source: Sci Rep. 2015 Jun 4;5:10855. doi: 10.1038/srep10855 (PMC4455149; doi:10.1038/srep10855)
Supplement: Supplementary Information [file srep10855-s1.doc]

**Supplementary Information**

**Electron spin coherence near room temperature in magnetic quantum dots**

Fabrizio Moro, Lyudmila Turyanska, James Wilman, Alistair J. Fielding, Michael W. Fay, Josef Granwehr, and Amalia Patanè.

**S1. W - band ESR spectrum**

**Figure S1 W-band CW-ESR experiment.** Experimental W-band CW ESR spectrum for a powder PbS:Mn sample (black curve) and simulations with cubic, forth order Stevenson operator *a*, (blue curve) and axial, second order Stevenson operator *D*, (red curve) symmetry, with *g*, *A* and$\Gamma_{\text{L}}$as in the main text and *D* strain of 15 MHz. The blue curve shows features, such as those indicated with an asterix (*) that strongly differ from the experimental spectrum; in contrast, the red curve shows an overall lineshape which resemble the experimental spectrum. Therefore, the simulation of the CW spectrum leans more towards a rombic character of the Mn local environment in the QDs.

**S2: Spin dynamics for Mn_0.05%_ in deuterated solvent matrices**

Figure S2 shows the echo decay traces for Mn_0.05%_ in different D_2_O-based solvent matrices. The dispersion of Mn_0.05%_ in D_2_O leads to a spin echo decay, magenta curve, ($T_{\mathrm{M}}$~ 1.1 µs), similar to that found for Mn_0.05%_ in H_2_O ($T_{\mathrm{M}}$ ~ 0.99 µs), which are both faster than the spin echo decay observed in powder samples ($T_{\mathrm{M}}$ ~ 1.6 µs), see discussion in the main text. The addition of protonated glycerol with ratios 1:1 and 1:3 (red and black curves) show longer spin echo decays ($T_{\mathrm{M}}$ ~ 1.9 µs and ~ 5.4 µs, respectively). The dispersion of Mn_0.05%_ in D_2_O/glycerol-D_8_ shows a further increased spin echo decay ($T_{\mathrm{M}}$~ 8.5 µs) modulated by deuterium spins hyperfine interaction. The apparent absence of proton modulation and the larger modulation depth of the deuterium frequency compared to the other solvent mixtures, suggest a larger deuterium spin density nearby the Mn ions possibly due to proximity of glycerol molecules to the QD’s surface and/or exchange of hydroxyl protons of the capping ligands with deuterium atoms.

Figure S2 Matrix dependent spin dynamics. Spin echo decay traces recorded for Mn_0.05%_ in different D_2_O-containing solvent matrices at *T* = 5 K and *B* = 345 mT.

**S3: Spin dynamics for Mn_0.01%_ as powder and frozen solution.**

Figure S3 shows that the spin echo decay for **Mn_0.01%_** in H_2_O/glycerol-H_8_ is slower than that for the powder sample. Also, the results of the fitting to a stretched mono-exponential decay function (equation 2 in the manuscript) show that $T_{\mathrm{M}}$ ~ 3.48 µs for **Mn_0.01%_** in frozen solution, which is similar to that for the sample **Mn_0.05%_** ($T_{\mathrm{M}}$ ~ 3.50 µs) dispersed in the same solvent. This result suggests that we have reached a limit of Mn concentration and hence Mn–Mn distances where Mn–Mn magnetic dipolar interactions are negligible.

**Figure S3. Spin echo decay for *x* = 0.01%.** Spin echo decay traces for **Mn_0.01%_** as powder (filled dots) and as frozen solution (empty dots). The red lines are fitting to equation 2 in the manuscript.

**S4: Spin echo temperature dependence for Mn_0.05%_ in H_2_O/glycerol-H_8._**

Figure S4 shows the temperature dependence of $T_{\mathrm{M}}$, 1/$T_{\mathrm{M}}$ and *s*, for **Mn_0.05%_** in H_2_O/glycerol-H_8_. 1/$T_{\mathrm{M}}$ depends linearly on temperature up to $T$ = 40 K. At higher temperature the echo signal drops below the noise level. We observe that *s* gradually decreases from ~ 1.8 at $T$= 3.2 K, reaching a plateau at ~ 1 for $T$ > 15 K.

Figure S4. Relaxation parameters for QDs in protonated water. Temperature dependence of $T_{\mathrm{M}}$ (spheres), 1/$T_{\mathrm{M}}$ (stars), and *s* for Mn_0.05%_ in H_2_O/glycerol-H_8_.

**S5: Echo field swept spectra**.

Figure S5(a) shows the echo field swept (EFS) spectrum measured for the powder **Mn_0.05%_** sample. The EFS spectrum is dominated by a peak centred at *g* ~2 and is modulated by six features. Fig. S5(b) shows the calculated first derivative of the EFS. In line with the interpretation of the CW-ESR spectrum discussed in the manuscript, we ascribe the observed peaks to transition between different *m_s_* and *m_I_* hyperfine states of Mn^2+^ ions encapsulated into **Mn_0.05%_** QDs with dominant contribution from the isotropic *m_s_* = ½ multiplet.

**Figure S5. Echo field swept spectrum.** EFS spectrum (a) measured for a powder **Mn_0.05%_** sample and its numerically calculated first derivative (b).

**S6: Temperature dependence of the spin dynamics.**

a)

b)

**Figure S6. Temperature dependent spin echo and inversion recovery traces.** Spin echo decay (a) and inversion recovery (b) traces for **Mn_0.05%_** in D_2_O/glycerol-D_8_ at different temperatures and fits to the data (black line) to a mono-and bi-exponential decay functions for (a) and (b), respectively. The estimates for $T_{\mathrm{M}}$, $T_{1}$ and $T_{\mathrm{SD}}$ as function of the temperature are reported in Table SII and Fig. 5 in the main text.

**Table SI Relaxation time parameters for *x* = 0.01%.** Results of fitting to the spin echo decay and inversion recovery data for **Mn_0.01%_** using equations 2 and 3 in the main text.

| **Mn_0.01%_** | **π**  **(ns)** | **τ**  **(ns)** | ***T*_M_ (µs)** | ***s*** | ***T*_1_ (µs)** | ***T*_SD_**  **(µs)** |
| --- | --- | --- | --- | --- | --- | --- |
| **Powder** | 120 | 210 | 2.22 ± 0.01 | 1.00 ± 0.01 | 332 ± 1 | 30 ± 0.5 |
| **H_2_O/glycerol-H_8_** | 32 | 400 | 3.48 ± 0.02 | 1.45 ± 0.02 | - | - |

**Table SII Relaxation time constants.** Results of the fitting of the data reported in Fig. S6 for **Mn_0.05%_** in D_2_O/glycerol-D_8_

| ***T* (K)** | ***T*_M_ (µs)** | ***T*_1_ (µs)** | ***T*_SD_ (µs)** |
| --- | --- | --- | --- |
| 4.5 | 8.19 ± 0.02 | 9790 ± 50 | 1034 ± 10 |
| 5 | 7.97 ± 0.01 | 8440 ± 70 | 1230 ± 12 |
| 8 | 6.61 ± 0.04 | 7300 ± 200 | 758 ± 10 |
| 10 | 6.51 ± 0.05 | - | - |
| 15 | 7.58 ± 0.08 | - | - |
| 20 | 6.98 ± 0.07 | 1830 ± 40 | 50 ± 3 |
| 25 | 6.7 ± 0.2 | - | - |
| 30 | 5.44 ± 0.08 | 308 ± 10 | 18 ± 2 |
| 40 | 5.1 ± 0.1 | 24 ± 6 | 4.4 ± 0.8 |
| 50 | - | 21 ± 5 | 3.4 ± 0.4 |
| 60 | 5.5 ± 0.2 | - | - |
| 80 | 4.5 ± 0.2 | 8.7 ± 2 | 1.7 ± 0.2 |
| 100 | 4.4 ± 0.2 | 3.1 ± 0.5 | - |
| 150 | 3.2 ± 0.3 | 6.1 ± 0.6 | - |
| 200 | 2.2 ± 0.4 | - | - |
| 230 | 1.3 ± 0.5 | - | - |
